# Efficacy of Plasma Exchange in Secondary Thrombotic Microangiopathy: A Case Report and Literature Review

**DOI:** 10.7759/cureus.100622

**Published:** 2026-01-02

**Authors:** Shohei Fukunaga, Naohito Masuda, Yudo Tanno, Takashi Yokoo

**Affiliations:** 1 Nephrology and Hypertension, The Jikei University Katsushika Medical Center, Katsushika-Ku, JPN; 2 Nephrology and Hypertension, The Jikei University School of Medicine, Minato-Ku, JPN

**Keywords:** infection, plasma exchange therapy, secondary thrombotic microangiopathy, systemic lupus erythematosus, thrombotic thrombocytopenic purpura

## Abstract

Thrombotic microangiopathy (TMA) is a severe syndrome characterized by thrombocytopenia, microangiopathic hemolytic anemia, and organ damage. It includes primary forms such as thrombotic thrombocytopenic purpura (TTP) and atypical hemolytic uremic syndrome, as well as secondary TMA, which can result from conditions such as autoimmune diseases, infections, and malignancies. While plasma exchange (PE) is a well-established treatment for thrombocytopenic purpura, its effectiveness against secondary TMA is debated, primarily because of the varying etiologies and pathomechanisms. There is a lack of comprehensive studies on the effectiveness of PE based on the underlying causes of secondary TMA. Herein, we report a woman in her 60s who was undergoing treatment for systemic lupus erythematosus (SLE). She presented with lower back pain and altered consciousness. The patient was diagnosed with a urinary tract infection and severe thrombocytopenia (1,000/μL). A PLASMIC score of 6 indicated a high likelihood of TTP, prompting the initiation of PE, which improved her platelet count. Her A disintegrin and metalloproteinase with thrombospondin motif 13 activity was 16%, confirming secondary TMA. The efficacy of PE for secondary TMA remains debated, with its effectiveness likely varying depending on the underlying cause. Case reports suggest poor outcomes for TMA caused by malignancies, whereas other causes tend to be associated with more favorable prognoses. However, no study has specifically examined the effectiveness of PE based on the primary underlying condition, highlighting the need for further research.

## Introduction

Thrombotic microangiopathy (TMA) is a syndrome characterized by thrombocytopenia, microangiopathic hemolytic anemia, and organ damage caused by platelet thrombi [1]. It can be classified into three categories: TMA caused by severely reduced A disintegrin and metalloproteinase with thrombospondin motif 13 (ADAMTS13) activity; TMA involving complement; and secondary TMA, which results from various underlying conditions. Secondary TMA can be triggered by autoimmune diseases, hematopoietic stem cell transplantation, organ transplantation, malignancies, pregnancy, infections, and medications [2]. Treatment for secondary TMA primarily targets the underlying cause, though plasma exchange (PE) may also be used [3]. In the present case, the patient developed TMA owing to medication use, an infection, and systemic lupus erythematosus (SLE). PE was performed, leading to an improvement in the platelet count. The efficacy of PE for secondary TMA remains debated; furthermore, no study has specifically examined the efficacy of PE for each cause, preventing a definitive conclusion about its overall effectiveness. To address this gap, we have summarized and analyzed past case reports on the use of PE for secondary TMA.

## Case presentation

A 69-year-old woman, diagnosed with SLE and lupus nephritis at 27 years of age, had been undergoing treatment. The patient’s medical history was long-standing; consequently, some details regarding her presentation were unclear. However, based on her records, she was diagnosed with SLE at the age of 27 years following the appearance of a skin rash. Subsequently, since proteinuria was detected, a renal biopsy was performed, leading to a diagnosis of lupus nephritis. During the course of the disease, there was no significant involvement of the other organs. Her renal function remained stable with a serum creatinine level of approximately 0.7 mg/dL, and urinalysis findings were unremarkable. One month before presentation, her regimen included prednisolone (5 mg/day), tacrolimus hydrate (3 mg/day), mycophenolate mofetil (500 mg/day), hydroxychloroquine sulfate (200 mg/day), esomeprazole magnesium hydrate (20 mg/day), sodium ferrous citrate (50 mg/day), and minodronic acid hydrate (50 mg) once every four weeks. Five days before admission, she developed lower back pain and mobility issues. Three days prior to presentation, she experienced altered consciousness and was transported to Hospital A. A computed tomography scan revealed a right ureteral stone, right hydronephrosis, and enlargement of the right kidney with surrounding fat stranding, leading to a diagnosis of calculous pyelonephritis. Stent placement and antibiotic therapy were initiated. On the day of admission, the patient was transferred to our hospital with the following vital signs: blood pressure, 137/80 mmHg; heart rate, 82 beats/min; body temperature, 38.4°C; and SpO2, 95% (on 2 L/min O2). She had a fever and exhibited altered consciousness with a Glasgow Coma Scale score of E2V2M4. Magnetic resonance imaging (MRI) of the head revealed multiple acute cerebral infarctions in the bilateral semioval centers, bilateral temporal lobes, basal ganglia, and right cerebellar peduncle. Blood tests showed renal dysfunction, high lactate dehydrogenase (LDH) level, and severe platelet count reduction to 1000/μL (Tables 1-2). 

**Table 1:** Urinalysis RBC, red blood cells; WBC, white blood cell

Parameters	Result	Reference range (unit)
Specific gravity	1.014	1.006-1.022
protein	1+	-
Occult blood	3+	-
RBC	Too many	-
WBC	30-49	-
Protein	5.17	<0.15 (g/gCr)

**Table 2:** Complete blood count and blood chemistry ADAMTS13, A disintegrin and metalloproteinase with thrombospondin motif 13; Alb, albumin; Alp, alkaline phosphatase; ALT, alanine aminotransferase; ANA, anti-nuclear antibody; anti-dsDNA Ab, anti-double stranded DNA antibody; APTT, activated partial thromboplastin time; AST, aspartate aminotransferase; BUN, blood urea nitrogen; C3, complement component 3; C4, complement component 4; Ca, calcium; CH50, hemolytic complement activity; Cl, chloride; Crea, creatinine; CRP, C-reactive protein; Hb, hemoglobin; Ht, hematocrit; IgA, immunoglobulin A; IgG, immunoglobulin G; IgM, immunoglobulin M;  K, potassium; LDH, lactate dehydrogenase; Lymph, lymphocytes; Mono, monocyte; Na, sodium;  N/A, not availableNAG; N-acetyl-β-D-glucosaminidase; Neut, neutrophil; P, phosphorus; P/C ratio, spot urine protein to creatinine ratio; PCT, procalcitonin; PG, plasma glucose; Plt, platelet; PT-INR, prothrombin time-international normalized ratio; RBC, red blood cells; T-bil, total bilirubin; TP, total protein; UN, urea nitrogen; WBC, white blood cell; β2-m, β2-microglobulin; γ-GTP, gamma-glutamyl transferase

Parameters	Result at admission	Result at the discharge	Reference range (unit)
WBC	14530	5400	3300-8860 (/μL)
Neut	95.5	40.7	40.0-75.0 (%)
Lynph	0.5	47	16.5-49.5(%)
Mono	4	9.6	2.0-10.0 (%)
RBC	3.91	3.77	386-492 (x10^6^/μL)
Hb	12	12.2	11.6-14.8 (g/dL)
Plt	10.5	18.6	15.8-34.8 (x10^4^/μL)
TP	6.8	7.3	6.6-8.1 (g/dL)
Alb	3.6	3.7	4.1-5.1 (g/dL)
T-bil	0.1	0.3	0.5-1.5 (mg/dL)
AST	24	21	13-30 (U/L)
ALT	17	15	7-23 (U/L)
LDH	238	305	124-222 (U/L)
γ-GTP	7	82	9-32 (U/L)
Alp	319	152	106-322 (U/L)
BUN	100.9	23	8-20 (mg/dL)
Crea	12.46	0.8	0.46-0.79 (mg/dL)
Na	124	140	138-145 (mmol/L)
K	7.8	5	3.6-4.8 (mmol/L)
Cl	88	105	101-108 (mmol/L)
Ca	8.9	9.3	8.8-10.1 (mg/dL)
P	6.2	3.7	2.7-4.6 (mg/dL)
CRP	6.38	0.81	< 0.14 (mg/dL)
IgG	993	N/A	861-1747 (mg/dL)
IgM	31	N/A	50-269 (mg/dL)
IgA	258	N/A	93-393 (mg/dL)
C3	66	87	86-160 (mg/dL)
C4	17	15	17-45 (mg/dL)
CH50	30	46.6	25.0-48.0 (U/mL)
ANA	1:40	1:40	<40
anti-dsDNA	11	20	<12.0 (IU/mL)
PT-INR	1	0.87	
APTT	29.4	23.5	23-40 (sec)
Fib	646	N/A	150-400 (mg/dL)
D dimer	0.6	N/A	<1.0 (μg/mL)
ADAMTS13 activity	16	N/A	>10 (%)
ADAMTS13 inhibitor	-	N/A	-

Given her severe infection status, antibiotics were switched from ampicillin sodium/sulbactam sodium to tazobactam/piperacillin, and hydrocortisone 400 mg/day was initiated. With a PLASMIC Score of 6, thrombotic thrombocytopenic purpura (TTP) was suspected, and PE was performed on day 3. Three sessions of PE were performed, each exchanging 1.2 total plasma volumes, with fresh frozen plasma (FFP) as the replacement fluid. After PE, her platelet count improved. However, fragmented red blood cells, low haptoglobin level, elevated LDH level, and decreased hemoglobin level were noted. The ADMATS13 activity was 16%, and no ADAMTS13 inhibitors were detected, confirming secondary TMA.* Klebsiella pneumoniae* was detected in both urine and blood cultures, prompting a switch to ceftriaxone on day 7. On day 7, prednisolone was reinitiated at 10 mg/day. Tacrolimus was discontinued owing to reports of medicine-induced TMA. Mycophenolate mofetil (500 mg/day) and hydroxychloroquine (200 mg/day) were restarted. During her hospital stay, she developed a persistent low-grade fever. Given her previous history of Cytomegalovirus (CMV) infection and ongoing use of immunosuppressive therapy, testing for CMV pp65 antigen was performed, yielding positive results. She also experienced a recurrent urinary tract infection, which was treated with valacyclovir and additional antibiotics. Consequently, the prednisolone dose was tapered to 7.5 mg/day. Her condition stabilized, and she was discharged on day 89.

## Discussion

TMA is a syndrome characterized by a triad of thrombocytopenia, microangiopathic hemolytic anemia, and organ damage due to platelet thrombi. Pathologically, it involves thrombosis and endothelial damage at the arteriolar and capillary levels. TMA is classified according to etiology, with secondary TMA resulting from an underlying disease. Known causes of secondary TMA include autoimmune diseases, hematopoietic stem cell transplantation, organ transplantation, malignancies, pregnancy, and medications.

In our patient, the ADAMTS13 activity was 16%, leading to a diagnosis of secondary TMA rather than TTP. Possible causes included SLE, tacrolimus-induced TMA, and infection. SLE is the most common underlying disease associated with collagen disease-related TMA [4], and it has been reported to occur simultaneously with disease activity [5]. In this case, the patient showed proteinuria, decreased CH50, and thrombocytopenia, with a SLEDAI score of 7, indicating active SLE. Furthermore, as the patient was taking tacrolimus, the possibility of medication-induced TMA could not be ruled out. 

Medication-induced TMA can be categorized into two types: one with significantly reduced ADAMTS13 activity, leading to secondary TTP, and one without significantly reduced ADAMTS13 activity, leading to atypical hemolytic uremic syndrome (aHUS) or secondary TMA. Additionally, there are two mechanisms: one being immunological, and the other, dose-dependent. For tacrolimus-induced TMA, no reduction in ADAMTS13 activity has been reported, and its onset is dose-dependent [6,7]. The incidence is low, approximately 1%-4.7%, predominantly in patients undergoing transplantation, and often occurs within one year of starting the medication. Treatment involves discontinuation of the drug or administration of PE [7]. Our patient had been taking tacrolimus for a long time without developing TMA; thus, it was not immediately suspected as the cause. However, as tacrolimus could not be definitively ruled out as the cause, the medication was discontinued. Additionally, *K. pneumoniae* infection could have played a role. Although rare, secondary TMA has been associated with *K. pneumoniae *infection [8-10]. *K. pneumoniae *is a neuraminidase producer, which may contribute to TMA development [11], though the detailed mechanism remains unclear. In this case, given the co-occurrence of SLE, medication use, and infection, determining the exact cause of TMA was challenging.

The efficacy of PE for secondary TMA remains uncertain, but its effectiveness for TTP is well-established [12]. Delayed initiation of PE worsens prognosis in TTP [13], indicating the importance of early intervention. However, the diagnosis of TTP or secondary TMA is often not immediately possible. Therefore, if TTP is suspected based on scores such as the PLASMIC and FRENCH scores, PE should be considered. In this case, the PLASMIC score suggested the possibility of TTP, leading to the initiation of PE, which resulted in improvement.

Treatment for secondary TMA varies depending on the underlying cause, but, as in this case, PE is sometimes used. PE functions through two primary mechanisms: the removal of pathogenic substances and the replenishment of normal components. Pathogenic substances include ADAMTS13 inhibitors, ultra-large von Willebrand factor multimers, and inflammatory cytokines, all of which can be cleared by PE. Normal components such as ADMATS13 and the normal von Willebrand factor can be replenished through this process. Although the efficacy of PE for TTP is well-established, opinions are divided regarding its use for secondary TMA. Some studies suggest that PE is effective even without severe ADAMTS13 deficiency [14], while others indicate that treatment can be safely managed without PE [15]. One study found no benefit of PE in patients with secondary TMA with ADAMTS13 activity above 10% [16]. However, these reports did not separate TMA causes for analysis. As the development of secondary TMA can be attributed to various causes and mechanisms, it is difficult to conclude that PE is uniformly ineffective. Therefore, further investigations into the efficacy of PE for each specific cause are necessary. 

To address the abovementioned gap, we reviewed and summarized case reports on secondary TMA treated with PE published on PubMed up to April 30, 2025. We analyzed 50 case reports, encompassing a total of 61 patients (Table 3). The mean age was 44.3 ± 22.5 years, with 25 male and 36 female patients. 

**Table 3:** Characteristics of the reviewed population ADAMTS13, A disintegrin and metalloproteinase with thrombospondin motif 13; F, female; M, male

Patient characteristics	
Total reference papers (n)	50
Age	44.3 ± 22.5
Sex (n)	
M	25
F	36
Platelet (/μL)	46900 ± 32157
Hemoglobin (g/dL)	7.9 ± 2.1
Serum creatinine (mg/dL)	3.9 ± 5.0
Lactate dehydrogenase (U/L)	1788 ± 1344
ADAMTS-13 (%)	58.2 ± 25.3
Number of plasma exchanges	9.2 ± 8.0
Outcome	
Improve	53
Not improve	2
Death	6

The most common cause was medication (11 cases), followed by malignancy (10 cases), acute pancreatitis (nine cases), infection (eight cases: seven cases of bacterial infection and one case of viral infection), and SLE (seven cases) (Table 4). In cases involving an unknown underlying disease, primary TMA was excluded, and the patient was diagnosed with secondary TTP of unknown etiology. Patients presented with low platelet counts (46,900 ± 32,156/μL), anemia (Hb: 7.9 ± 2.1 g/dL), high LDH levels (1788 ± 1345 U/L), and renal dysfunction (serum creatinine: 7.9 ± 2.1 mg/dL). PE was performed an average of 9.2 ± 8.0 times. Concomitant treatments included oral steroids (13 patients), eculizumab (seven cases), steroid pulse therapy (two cases), rituximab (two cases), and no concomitant treatments (27 patients). Other cases involved combinations of these treatments. Outcomes showed improvement in 53 cases, no improvement in two cases, and death in six cases. The malignancy group had the highest proportion of poor outcomes (death or no improvement), occurring in four (death, three cases; no improvement, four cases) out of 10 cases. Death was also observed in one case each from the following groups: postoperative infection, acute pancreatitis, and unknown causes. No improvement was also observed in one case each from the following groups: SLE, mixed connective tissue disease, and Sjögren's syndrome. Overall, malignancy was associated with the poorest prognosis. 

**Table 4:** Primary disease of secondary thrombotic microangiopathy

Cause	
Drug	11
Cancer	10
Acute pancreatitis	9
Systemic lupus erythematosus	7
Infection	8
Postoperative	3
Systemic scleroderma	2
Pregnancy	2
Adult onset Still's disease	1
Juvenile rheumatoid arthritis	1
Secondary sclerosing cholangitis	1
Mixed connective tissue disease + Sjögren's syndrome	1
Antiglomerular basement membrane	1
Sickle cell disease	1
Trauma	1
Unknown	2

The mechanisms of malignancy-induced TMA can be broadly categorized into two types: those caused by the cancer itself and those caused by anticancer chemotherapy [17]. Cancer-induced mechanisms include direct tumor cell infiltration into microvessels, endothelial cell damage, and the release of ultra-large von Willebrand factor multimers and inflammatory cytokines [18]. Although the removal of ultra-large von Willebrand factor multimers and inflammatory cytokines is possible with PE, it cannot effectively address tumor cell infiltration. Therefore, the effectiveness of PE in treating malignancy-induced TMA may be limited. However, as this study involved a review of previously published case reports, caution is warranted when interpreting the findings due to the possibility of publication bias, and prospective studies are needed in the future. 

## Conclusions

This study indicates that the effectiveness of PE in treating secondary TMA varies depending on the underlying cause. Analysis of case reports showed malignancy-induced TMA has a poorer prognosis, while other causes generally respond better to PE. Although PE is established for TTP, its benefit in secondary TMA remains uncertain and likely depends on the pathophysiology. Early initiation of PE may improve outcomes, but further research is needed to clarify its efficacy specifically according to each underlying condition.
